# Intra-arterial chemotherapy for treatment of very low birthweight infants with retinoblastoma: Two case reports

**DOI:** 10.1097/MD.0000000000025870

**Published:** 2021-05-14

**Authors:** Hua Jiang, Yizhou Jiang, Zhenyin Liu, Yiqun Guo, Jing Zhang

**Affiliations:** a Department of Interventional Radiology and Vascular Anomalies, The Affiliated Hospital of Guangzhou Medical University, Guangzhou Women and Children's Medical Center; b Department of Interventional Radiology, Guangdong Provincial People's Hospital, Guangdong Academy of Medical Sciences, Guangzhou, China.

**Keywords:** Chemotherapeutic agents, complications, Intra-arterial chemotherapy, very low birthweight

## Abstract

**Introduction:**

Intra-arterial chemotherapy (IAC) has been used successfully for unilateral and bilateral eye disease with advanced or less advanced intraocular disease and of children as young as 3 months of age. In this study, we expanded the use of IAC to eyes of two infants with very low birthweight (birth weight of 1000–1500 g) and retinoblastoma.

**Patient concerns:**

The ophthalmologists refused to give patients intravenous chemotherapy (IVC), considering the risk of IVC because of the low body weight.

**Diagnosis:**

Two patients were diagnosed unilateral eye retinoblastoma at 31 days old and 27 days old respectively when newborn fundus screening.

**Interventions:**

Chemotherapeutic agents were injected into the ophthalmic artery during the four IAC session. The two patients’ oculars were preserved due to calcification during follow-up periods to the final IAC.

**Outcomes:**

Complications included eyelid edema in the patient #1 and neutropenia in both patients. No fever, sepsis and other systemic toxic effects were observed. After two successful IAC treatment for each patient, their oculars were all preserved without serious complications.

**Lessons:**

Our center for the first time reported the management of retinoblastoma in very low birthweight infants with especially challenging and we recommend this to be useful to the patient community.

## 1 Introduction

Intra-arterial chemotherapy (IAC) has been successfully used for unilateral and bilateral eye disease with advanced or less advanced intraocular disease and in children as young as 3 months old. However, side effects, advantages, and disadvantages of IAC have been described in various reviews.^[1,2]^ Moreover, it is even more difficult to apply IAC in children with very low birthweight (VLBW, birth weight of 1.0–1.5 kg), due to the slow growth and slender blood vessels. In this study, we expanded the use of IAC for retinoblastoma in two infants with very low birthweight.

## 2 Case report

### 2.1 Case #1

The patient #1 was a girl and her mother gave birth to her at 40 weeks of pregnancy with spontaneous delivery and the birth weight was 1.1 kg. The patients was diagnosed with unilateral eye retinoblastoma (International Classification Group B) at 31 days old during newborn fundus screening. No leucocoria, strabismus or other clinical presentation were appeared. The ophthalmologists refused to give the patient intravenous chemotherapy (IVC), considering the risk of IVC because of the low body weight. Then the patient #1 received IAC for the first session at 45 days old with only 1.75 kg and the second session at 72 days old with 2 kg.

The femoral artery was punctured with a puncture needle of 20 G instead of a 4-French (4-F) arterial sheath using Seldinger technique. After successful puncture, the needle sheath was retained in the femoral artery and then connected with Angioplasty Packs (Merit Medical Systems, Inc, South Jordan, USA). Heparin (75 IU/kg) was administered by intravenous injection to avoid thrombosis. Under X-ray guidance, the ipsilateral internal carotid artery was catheterized with 1.5F ev3 45° microcatheter (v3 Neurovascular, Inc., Irvine, CA, USA) guided by a micro-guide wire. After the path of the ophthalmic artery from the internal carotid artery was determined, the ophthalmic artery was catheterized by using fluoroscopy and road mapping. When the microcatheter was placed at the ostium of the ophthalmic artery, an angiogram of the ophthalmic artery was taken. The chemotherapeutic agents included melphalan (0.8 mg) and carboplatin (20 mg) used in the first session of IAC and melphalan (0.8 mg) and topotecan (0.5 mg) in the second session. At the conclusion of the infusion, the microcatheter was withdrawn and the sheath was removed. Hemostasis of the femoral artery was then achieved by manual compression for 10–15 min.

The treatment of results including treatment features and complications are listed in Table 1. Chemotherapeutic agents were all successfully injected into the ophthalmic artery during the four IAC session. The patient's oculars were preserved due to calcification during follow-up periods to the final IAC (Figure 2). Complications including eyelid edema and neutropenia in the patient #1 was observed. No fever, sepsis and other systemic toxic effects were observed.

**Table 1:** Results of IAC for retinoblastoma in two infants with very low birthweight.

N	Family history	Age & weight at First IAC	Age & weight at second IAC	Other treatments	Follow-up (months)	Complications
1	no	45 days & 1.75 kg	72 days & 2.0 kg	none	15	eyelid edema, neutropenia
2	no	50 days & 1.8 kg	78 days & 2.5 kg	laser	14	neutropenia

IAC: Intra-arterial chemotherapy.

### 2.2 Case #2

**Figure 1:**
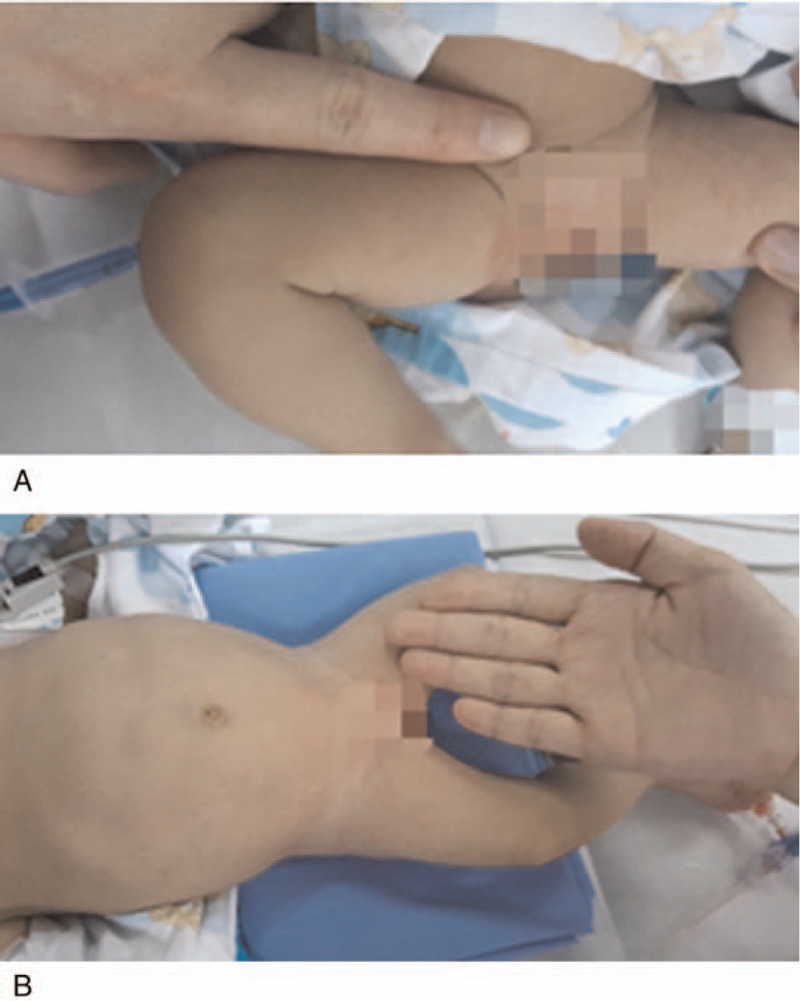
Diameter comparison of one of operator's finger to the thigh of a child. (A & B) The fingers were from a male operator of weight 65 kg, height of 175 cm. The thigh was from the patient #2 whose weight was 2.5 kg at the second session of IAC treatment. The diameter of the thigh of the patients was almost equivalent to the sum of the diameters of the three fingers.

The patient #2 was also a girl born at 31 weeks gestation with cesarean section and the birth weight was 1.2 kg. The patient was diagnosed with unilateral eye retinoblastoma (International Classification Group B) at 27 days old, respectively during newborn fundus screening. No leucocoria, strabismus or other clinical presentation were appeared. The ophthalmologists refused to give the patient intravenous chemotherapy (IVC), considering the risk of IVC because of the low body weight. The patient #2 received the first session of IVC at 50 days old with only 1.8 kg and the second session at 78 days old with 2.5 kg (Figure 1) in addition to once focal laser treatment.

The femoral artery was punctured with a puncture needle of 20 G instead of a 4-French (4-F) arterial sheath using Seldinger technique. After successful puncture, the needle sheath was retained in the femoral artery and then connected with Angioplasty Packs (Merit Medical Systems, Inc, South Jordan, USA). Heparin (75 IU/kg) was administered by intravenous injection to avoid thrombosis. Under X-ray guidance, the ipsilateral internal carotid artery was catheterized with 1.5F ev3 45° microcatheter (v3 Neurovascular, Inc., Irvine, CA, USA) guided by a micro-guide wire. After the path of the ophthalmic artery from the internal carotid artery was determined, the ophthalmic artery was catheterized by using fluoroscopy and road mapping. When the microcatheter was placed at the ostium of the ophthalmic artery, an angiogram of the ophthalmic artery was taken. The chemotherapeutic agents included melphalan (0.8 mg) and carboplatin (20 mg) used in the first session of IAC and melphalan (0.8 mg) and topotecan (0.5 mg) in the second session. At the conclusion of the infusion, the microcatheter was withdrawn and the sheath was removed. Hemostasis of the femoral artery was then achieved by manual compression for 10–15 min.

**Figure 2:**
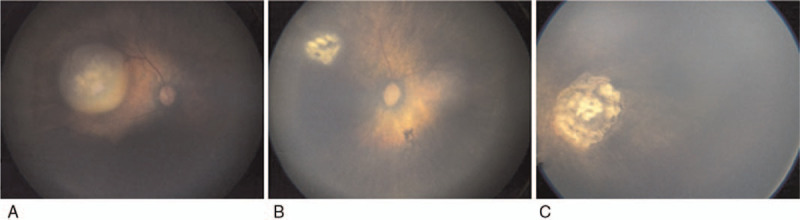
Intra-arterial chemotherapy (IAC) for infants of very low birthweight. (A) A 45-day-old girl (patient #1) with unilateral sporadic retinoblastoma in left eye. (B) Fundus photographs after 2 cycles of IAC. After 2 cycles of IAC, complete tumor regression was achieved. (C) After 15 months of follow-up, no recurrence of the tumor was observed.

The treatment of results including treatment features and complications are listed in Table 1. Chemotherapeutic agents were all successfully injected into the ophthalmic artery during the four IAC session. The patient's oculars were preserved due to calcification during follow-up periods to the final IAC (Figure 2). Complications including neutropenia in the patient #2 were observed. No fever, sepsis and other systemic toxic effects were observed.

## 3 Discussion

IAC was an invasive technique that limited its clinical application in young infants, due to the potential risk of complications from catheterizing small arteries.^[2]^ Although we have reported performing IAC in 10 young infants less than 3 months old,^[1]^ no other center has reported the management of retinoblastoma in very low birthweight infants that is particularly challenging, which is why we believe it is important to share our experience. After two successful IAC treatments for each patient, their oculars were all preserved without serious complications.

For these two patients, at first, we have hesitated to treat such low-weight children less than 2 kg with IAC. Higher frequency of systemic side effects and less effective in children younger than 2 months old with IVC^[4]^ have led ophthalmologists abandon IVC treatment for the two patients. Considering lower growth rate and lower tolerance to chemotherapy of VLBW infants than the normal birth weight infants,^[5]^ it may take a longer time for these children to grow to 6 kg by using the bridge IV-IA chemotherapy which used IVC to postpone IAC until children reach the age of 3 months and a weight of 6 kg. After careful weighing and the demands of children's parents, we finally decided to use IAC technology for treatment.

The biggest challenge of IAC treatment for infants is how to avoid technical complications resulted from the procedure, since the small diameter of the blood vessel is just slightly larger than the catheters. We puncture the femoral artery with a 20G puncture needle instead of the 4F arterial sheath for children, reducing in the size of the femoral access sheath to 0.8 mm diameter instead of the usual 1.3 mm. The previously reported IAC treatment of retinoblastoma in young infants using a microcatheter (1.5 F or 0.5 mm distal diameter) gave us confidence in treating the two patients.^[1]^ In this report, we report no complaints of catheterization associated complications.

The main drug for IAC was melphalan which may induce significant neutropenia when given at a dose higher than 0.5 mg/kg.^[3]^ The tricky thing is that drug dose for these patients is far more than 0.5 mg/kg even with the smallest eye dose of melphalan as previously described according to clinical and anatomical factors (ranged from 2.5–3 mg). Firstly, to achieve the best effect without serious complications, we administered melphalan a total dose of 0.8 mg and no more than a dose of 0.5 mg/kg. Secondly, we used double drug IAC for using smaller doses of melphalan combined with carboplatin or topotecan to avoid the system toxicity of single drug chemotherapy.^[1]^ After IAC treatment, both patients had Grade 3–4 neutropenia, with or without symptomatic treatment. The two patients’ oculars were preserved due to calcification during follow-up periods.

The findings of this study suggest that IAC is effective and fairly safe for the management of advanced retinoblastoma in infants with low body weight and high technical success rate. Despite these favorable initial results, adverse events related to intra-arterial injection and the visual outcomes cannot be neglected and requires further investigation. Until the precise benefits and risks of IAC are identified, this treatment should be used with caution in very young infants.

## 4 Conclusions

Serious complications are the main obstacle in the IAC treatment for infants. In this study, we expanded the use of IAC to eyes of two children with very low birthweight. IAC treatment may be considered in the infants with retinoblastoma in the clinical practice.

## Author contributions

**Conceptualization:** Jing Zhang.

**Data curation:** Hua Jiang, Jing Zhang.

**Funding acquisition:** Jing Zhang.

**Investigation:** Hua Jiang, Jing Zhang.

**Methodology:** Zhenyin Liu.

**Project administration:** Yizhou Jiang.

**Validation:** Yizhou Jiang, Zhenyin Liu.

**Writing – original draft:** Jing Zhang.

**Writing – review & editing:** Yiqun Guo.
